# Monoclonal Antibody Combinations Prevent Serotype A and Serotype B Inhalational Botulism in a Guinea Pig Model

**DOI:** 10.3390/toxins11040208

**Published:** 2019-04-06

**Authors:** Milan T. Tomic, Yero Espinoza, Zachary Martinez, Khanh Pham, Ronald R. Cobb, Doris M. Snow, Christopher G. Earnhart, Traci Pals, Emily S. Syar, Nancy Niemuth, Dean J. Kobs, Shauna Farr-Jones, James D. Marks

**Affiliations:** 1 Research and Development, Ology Bioservices, Inc., 626 Bancroft Way, Suite D, Berkeley, CA 94710, USA; yero.espinoza@ologybio.com (Y.E.); Zach.martinez@ologybio.com (Z.M.); khanh.pham@ologybio.com (K.P.); 2 Ology Bioservices, Alachua, FL 32615, USA; ron.cobb@ologybio.com (R.R.C.); doris.snow@ologybio.com (D.M.S.); 3 PRISM Program Office, Medical Countermeasures Systems, Ft. Detrick, MD 21702, USA; christopher.earnhart@navy.mil; 4 Vaccines and Therapeutics Division, Chemical and Biological Office, Ft. Detrick, MD 21702, USA; traci.k.pals.civ@mail.mil; 5 Battelle Biomedical Research Center, West Jefferson, Columbus, OH 43162, USA; syare@battelle.org (E.S.S.); niemuth@battelle.org (N.N.); kobsd@battelle.org (D.J.K.); 6 Department of Anesthesia and Perioperative Care, University of California, 1001 Potrero Ave., San Francisco, CA 94110, USA; Shauna.Farr-Jones@ucsf.edu (S.F.-J.); jim.marks@ucsf.edu (J.D.M.)

**Keywords:** botulinum neurotoxin, aerosol, monoclonal antibody, guinea pig inhalation model, oligoclonal antibody, inhalational botulism, botulism

## Abstract

Botulinum neurotoxins (BoNT) are some of the most toxic proteins known, with a human LD_50_ of ~1 ng/kg. Equine antitoxin has a half-life in circulation of less than 1 day and is limited to a treatment rather than a prevention indication. The development of monoclonal antibodies (mAbs) may represent an alternative therapeutic option that can be produced at high quantities and of high quality and with half-lives of >10 days. Two different three mAb combinations are being developed that specifically neutralize BoNT serotypes A (BoNT/A) and B (BoNT/B). We investigated the pharmacokinetics of the anti-BoNT/A and anti-BoNT/B antibodies in guinea pigs (*Cavia porcellus*) and their ability to protect guinea pigs against an aerosol challenge of BoNT/A1 or BoNT/B1. Each antibody exhibited dose-dependent exposure and reached maximum circulating concentrations within 48 h post intraperitoneal or intramuscular injection. A single intramuscular dose of the three mAb combination protected guinea pigs against an aerosol challenge dose of 93 LD_50_ of BoNT/A1 and 116 LD_50_ of BoNT/B1 at 48 h post antibody administration. These mAbs are effective in preventing botulism after an aerosol challenge of BoNT/A1 and BoNT/B1 and may represent an alternative to vaccination to prevent type A or B botulism in those at risk of BoNT exposure.

## 1. Introduction

The botulinum neurotoxins (BoNTs) produced by bacteria of the genus *Clostridium* are the most toxic proteins known [1,2]. Structurally, BoNTs are a family of 150 kDa endopeptidase protoxins, which are subsequently cleaved to generate a disulfide bond-linked dipeptide consisting of a 100 kDa heavy chain (HC), comprised of a binding domain H_C_ and a translocation domain H_N_ and a 50 kDa light chain (LC) [3,4]. The H_C_ binds receptors on the presynaptic membrane [5,6] leading to BoNT endocytosis. After endocytosis, the H_N_ forms a channel across the endosomal membrane allowing delivery of the LC into the cytoplasm [7,8,9]. The LC is a zinc-endopeptidase that cleaves SNARE proteins, thereby blocking synaptic vesicle fusion and acetylcholine release [10] resulting in failure of neuromuscular transmission.

BoNTs can be classified into at least seven serotypes (A–G) [11] defined immunologically by the inability of polyclonal IgG antibodies that neutralize one serotype to neutralize the other serotypes [12,13]. BoNT serotypes A, B, E, F cause the disease botulism in humans [14,15,16]. Botulism is a disease with four distinct, naturally occurring etiologies: foodborne, wound, infant botulism and adult intestinal botulism and a fifth etiology being iatrogenic botulism, for example caused by therapeutic administration of BoNT for cosmetic or movement disorders [17,18]. Botulism can also occur by the inhalation route [19,20] but there is only a single case report of naturally occurring inhalational botulism in laboratory workers [21]. Each of these etiologies result in similar clinical symptoms including symmetrical cranial nerve palsies followed by descending, symmetric, flaccid muscle paralysis of voluntary muscles which may progress to respiratory compromise and death [16,22]. Mechanical ventilator support is commonly needed for 2 to 8 weeks, with some patients requiring support for up to 7 months. Less than 200 cases of botulism occur annually in the US with the majority of the cases being infant botulism and caused by serotypes A and B [14,16]. Botulism cases caused by BoNT/A, B and E have also been reported worldwide [23,24,25]. BoNT/F causes only 1% of food poisoning-related cases of botulinum intoxication [26].

BoNTs are classified as Tier 1 biothreat agents by the Federal Select Agent Program of the Centers for Disease Control and are among a limited number of biothreat agents with the highest risk of potential use as bioweapons due to their potency and routes of oral or inhalational exposure [22,27]. A human lethal dose of BoNT/A is estimated to be 0.7 μg by the inhalational route and 70 μg by the oral route [22]. Both Iraq and the former Soviet Union produced BoNT for use as weapons with intent for aerosol release [28,29] and the Japanese cult Aum Shinrikyo attempted to use BoNT for bioterrorism by dispersing toxin aerosols in Tokyo. Finally, the increased medicinal use of BoNTs [30] and their legal and illicit manufacture in countries such as China, Brazil and elsewhere in emerging economies makes BoNTs easily available to those of ill intent [31].

Current strategies to prevent botulism, including intentional or biothreat botulism, are focused primarily on vaccination. There is, however, no FDA licensed BoNT vaccine available. For decades, an investigational pentavalent vaccine (serotypes A-E) was available and distributed by the CDC for laboratory workers and the military but the vaccine was withdrawn from clinical use in 2011 due to lack of efficacy [32,33] resulting from the age of the product. A number of recombinant BoNT vaccines are under development with the most advanced a bivalent serotype A and B based on the H_C_ domain (rBV A/B) and expressed from *Pichia pastoris* [34,35]. rBV is being developed specifically for the Department of Defense’s Joint Vaccine Acquisition Program to protect warfighters against inhalational botulism [36]. The rBV A/B has undergone two Phase 1 and 1 Phase 2 clinical studies [36]. In the Phase 1 trials, more than 80% of volunteers developed neutralizing antibodies above the lower limit of detection to BoNT/A and BoNT/B and longer vaccination schedules elicited a greater neutralizing antibody titer than shorter schedules. In the Phase 2 trial using vaccination with 40 mg of total immunizing protein at 0, 28 or 56 and 182 days, the peak neutralizing antibody titer was at 210 days. Duration of protection has not been reported.

An alternative to vaccination is the prophylactic administration of neutralizing antibody or antitoxins which, compared to vaccination, would provide immediate immunity in all subjects dosed. Neutralizing antibody would need to be available in adequate quantities and have a half-life of weeks in order to provide the necessary protection. Two antitoxins are currently approved for treatment of botulism, human botulism immune globulin (BIG-IV) and equine-derived heptavalent botulinum antitoxin (BAT) [37,38,39,40]. Neither of these are vaccine alternatives. BIG-IV is used for the treatment of infant botulism and is produced by plasmapheresis of immunized human laboratory workers. The small quantities produced preclude its use for large scale passive immunization of adults [22]. BAT is approved by the FDA to treat non-infant botulism and is a Fab’_2_ antibody derived from immunized horses. BAT has associated hypersensitivity reactions including cardiac arrest and serum sickness and most importantly has a very short half-life (7.5–34.2 h depending on serotype). The short half-life precludes the use of BAT for prevention of botulism since protective serum levels of antibody are short lived, on the order of days [41].

As a potential alternative to BAT for the treatment of botulism, potent human monoclonal antibody (mAb) based antitoxins composed of three mAbs binding non-overlapping epitopes are being developed [42,43,44,45,46]. The most advanced of these products are the BoNT/A and BoNT/B antitoxins being developed, NTM-1631 and NTM-1632 respectively, which have completed Phase 1 testing in humans without serious adverse side effects [47]. NTM-1631 is an equimolar combination of three monoclonal antibodies (humanized mAb XA-a and human mAbs XA-b and XA-c) that bind and neutralize BoNT/A [47]. NTM-1632 is an equimolar combination of three monoclonal antibodies (human mAbs XB-a, XB-b, XB-c) that bind and neutralize BoNT/B [44].

To determine the ability of these recombinant antitoxins to be used as an alternative to vaccination with rBV for the prevention of inhalational botulism, we evaluated the ability of NTM-1631 and NTM-1632 to prevent BoNT/A and BoNT/B inhalational botulism in an aerosol challenge model in guinea pigs. Circulating antibodies reached a peak concentration between 12–72 h after the IM administration and completely protected guinea pigs challenged with up to 93 LD_50_ of BoNT/A1 toxin and 116 LD_50_ of BoNT/B1.

## 2. Results

### 2.1. Evaluation of Dose and Route of Administration on Individual mAb Pharmacokinetics and NTM-1631 and NTM-1632 Neutralizing Antibody Concentration (NAC) in Guinea Pigs

**Figure 1:**
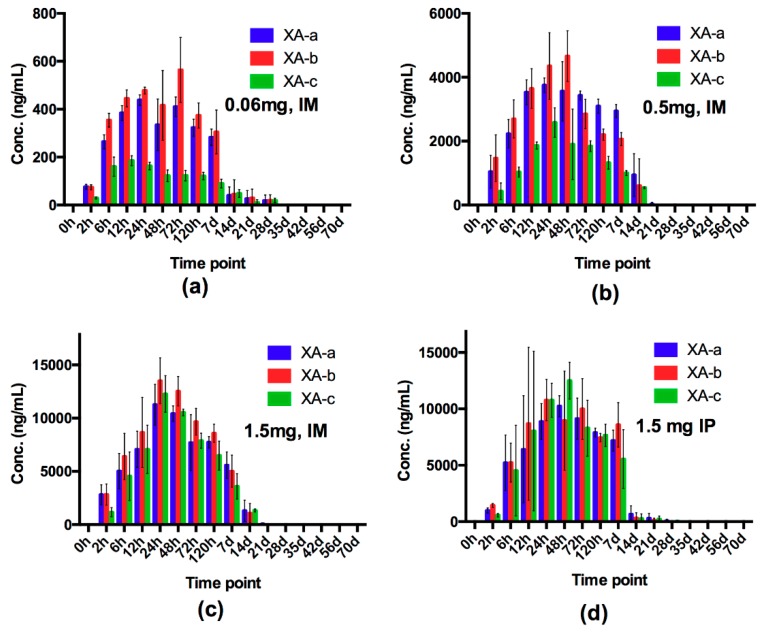
NTM-1631 antibody distribution in guinea pigs. A total of 192 male guinea pigs were administered NTM-1631 intramuscularly (IM) or intraperitoneally (IP) as described in the Materials and Methods. Each data point is an average of 2–4 animals, error bars indicate %CV. (**a**) IM 0.06 mg; (**b**) IM 0.5 mg; (**c**) IM 1.5 mg; (**d**) IP 1.5 mg.

**Figure 2:**
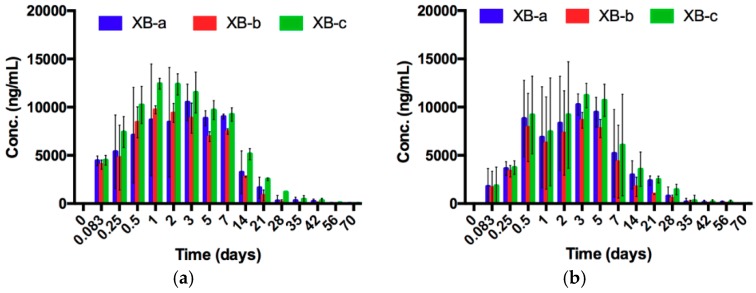
NTM-1632 Distribution in guinea pigs. A total of 192 male guinea pigs were administered NTM-1632 IM or IP as described in the Materials and Methods. Each data point is an average of 2–4 animals. Error bars indicate standard deviation. (**a**) IM 1.5 mg dose; (**b**) IP 1.5 mg dose.

To validate route of administration and determine timing of BoNT exposure, the concentration of each component mAb and the neutralizing antibody concentration (NAC) of NTM-1631 and NTM-1632 was evaluated in guinea pigs. The concentration of each component mAb was measured using previously developed recombinant BoNT/A and BoNT/B mAb specific domains that allow quantitation of each of the three component mAbs in NTM-1631 and NTM-1632 in vitro and in vivo [44,45]. The concentration of mAbs determined by the ECL assay showed that peak or near-peak mAb serum concentrations occurred 12 to 72 h after IM or IP injection of NTM-1631 (Figure 1) and NTM-1632 (Figure 2). Since the IP route is commonly used for antitoxin administration, we compared mAb serum concentration after IM administration to IP administration at the highest 1.5 mg dose. For both NTM-1631 and NTM-1632, the IP route of injection resulted in maximal mAb concentrations that appear to occur later than after IM injection (Figure 1c vs. Figure 1d and Figure 2a vs. Figure 2b). IP administration yielded comparable peak mAb concentrations as IM administration. For both NTM-1631 and NTM-1632, concentrations of the three component mAbs were relatively comparable at the 1.5 mg total injected doses (Figure 1c,d and 2a,b). In contrast, at the lower IM doses of 0.06 mg and 0.5 mg for NTM-1631, the serum concentration of mAb XA-c was lower than that of XA-a and XA-b). This difference was statistically significant (two-way ANOVA and multiple comparison *t*-test) for the 0.06 mg dose, F(2,6) = 228.5; *p* < 0.0001; and the 0.5 mg dose F(2,6) = 88.76, *p* < 0.0001 at the 6 h to 3 day timepoints. At all doses and time points, mAbs XA-a and XA-b were not significantly different from each other. The reason for the differences in mAb XA-c concentration at the 0.06 and 0.5 mg dose is not clear. However, because of the variability in mAb concentrations at the 0.06 mg and 0.5 mg doses of NTM-1631, we elected to evaluate NTM-1632 only at the 1.5 mg dose.

Serum mAb concentrations decreased relatively rapidly after 7–14 days. Since the human antibodies are a foreign protein in guinea pigs, it is possible that part of the decrease at 14 days and later time points was due to anti-drug antibodies (ADA), though we did not measure ADA levels.

**Figure 3:**
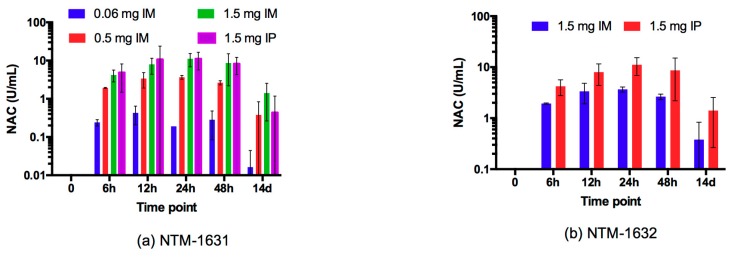
Neutralizing antibody concentrations (NAC) after injection of NTM-1631 or NTM-1632. Each data point is the mean of measurements from three animals administered the indicated amount of NTM-1631 or NTM-1632 IM or IP. (**a**) Neutralizing antibody concentrations after injection of NTM-1631. (**b**) Neutralizing antibody concentrations after injection of NTM-1632.

The neutralizing antibody concentration (NAC) of NTM-1631 and NTM-1632 in guinea pig serum was determined by the mouse neutralization assay (MNA) and expressed in Units (U)/mL where 1 U = 10,000 mouse intraperitoneal LD_50_s (MIPLD_50_s/mL). Serum from three individual guinea pigs per time point were tested in the MNA at 0, 6, 12, 24 and 48 h and 14 days, for a total of 18 serum samples per group. The NAC was determined for each animal and the average NAC per time point was calculated. Four groups were studied for NTM-1631, IM administration at NTM-1631 doses of 0.06 mg, 0.5 mg, 1.5 mg and IP administration at a dose of 1.5 mg. The NTM-1631 0 h samples were tested using the qualitative MNA for serotype A and all time 0 samples had no measurable levels of NAC (Figure 3a). At 6 h post NTM-1631 administration, all animals in the four groups had measurable NAC. With IM administration, NAC increased with increasing dose, with a peak NAC between 12 and 48 h, with peak NAC levels similar at these time points and with a peak of 11 U/mL at the 1.5 mg dose. NAC decreased to 1.4 U/mL or less at Day 14 for all NTM-1631 groups (Figure 3a).

Based on the NTM-1631 results, two groups were studied for NTM-1632, either IM or IP administration at a dose of 1.5 mg. The NTM-1632 0 h samples were tested using the quantitative MNA for serotype B and all time 0 samples had no measurable levels of NAC (Figure 3b). The average NAC increased for both groups with a peak at 24 h for IP and IM administration and then decreased. For IM administration, peak NAC at 24 h was 15 U/mL with a value of 10 U mL at 48 h (Figure 3b). At 14 days, NAC had decreased to approximately 9 U/mL, significantly higher than the values seen for NTM-1631 at 14 days.

### 2.2. NTM-1631 and NTM-1632 Protect Guinea Pigs against Lethal Aerosol Challenge with BoNT/A and BoNT/B

Two inhalational BoNT/A and BoNT/B challenge studies were conducted (Table 1). In the first guinea pig challenge study, a dose of 10 guinea pig LD_50_ for each of BoNT/A1 and BoNT/B1 toxins was targeted. As shown in Table 1, the guinea pigs actually received 55 guinea pig aerosol LD_50_ of BoNT/A and 8 guinea pig aerosol LD_50_ of BoNT/B as calculated from the amount of BoNT measured in the aerosol in LD_50_s and the measured total accumulated tidal volume in liters (TATV). In the second guinea pig challenge study, a target dose of 100 guinea pig aerosol LD_50_ for each of BoNT/A1 and BoNT/B1 toxins was selected by increasing the concentration of BoNT in the nebulizer suspension. As shown in Table 1, the guinea pigs received 93 guinea pig aerosol LD_50_ of BoNT/A1 and 116 guinea pig aerosol LD_50_ of BoNT/B1 as calculated as the product of the amount of BoNT measured in the aerosol in LD_50_s and the measured TATV.

To validate that sufficient neutralizing antibodies were present in the serum of the guinea pigs prior to challenge with BoNT, three guinea pigs were terminally bled on the day of BoNT challenge 48 h after IM administration of 1.5 mg each of NTM-1631 or NTM-1632. These samples were then analyzed using the MNA for the presence of neutralizing antibodies. As shown in Table 2, the animals injected with PBS had no measurable level of NAC. In contrast, animals injected with NTM-1631 showed NAC of 7.7 U/mL and 9.7 U/mL in Studies 1 and 2, respectively. Guinea pigs injected with the NTM-1632 demonstrated higher levels of NAC (21.6 U/mL and 19.5 U/mL in Studies 1 and 2, respectively) than those observed in animals injected with the NTM-1631 (Table 2 and Tables S1–S4). These results were consistent with results from the dose ranging studies (Figure 3).

For protection studies, 1.5 mg/guinea pig of NTM-1631 or NTM-1632 was administered intramuscularly 48 h prior to aerosol challenge (Table 3). All animals that were dosed with PBS died within 15 h post challenge with 55 guinea pig LD_50_ of BoNT/A1 or within 24 h post-challenge with 8 LD_50_ of guinea pig BoNT/B1 (Table 3). In the first challenge study, all animals that received NTM-1631 or NTM-1632 survived for 14 days (the duration of the study) post challenge with BoNT/A1 or B1.

A second challenge experiment was conducted with a target dose of 100 LD_50_ of BoNT/A1 or BoNT/B1. All animals that were dosed with PBS died within 13 h post challenge with 93 LD_50_ of BoNT/A1 or within 14 h post-challenge with 116 LD_50_ of BoNT/B1 (Table 3). All animals that received NTM-1631 or NTM-1632 survived for 14 days (the duration of the study) post challenge with BoNT/A1 or B1. In both challenge studies, no adverse clinical symptoms were observed in any of the animals in the antibody treated groups and all animals treated with antibody and challenged with BoNT gained weight during the course of the experiment further indicating no effect from the toxin exposure when toxin-specific antibodies were in present circulation.

## 3. Discussion

Current strategies to prevent botulism, including biothreat botulism, are focused primarily on vaccination. A recombinant vaccine is currently under development (rBV A/B) for the protection of warfighters against inhalational botulism due to BoNT/A1 and BoNT/B1 exposure [36,48,49]. A potential alternative to active vaccination is passive immunization achieved by administering a neutralizing antibody. Due to limitations in the amount produced, the small quantities of BIG-IV produced for the treatment of infant botulism preclude its use for the large scale passive immunization of adults [22]. BAT, used to treat adult botulism, is an Fab’_2_ antibody which has a very short half-life (7.5–34.2 h depending on serotype) that precludes its use for prevention of botulism [41]. Here we show that passive immunization with human or humanized mAb combinations is a viable alternative to active immunization for prevention of botulism. The three-mAb combinations (NTM-1631 and NTM-1632) completely protected guinea pigs against lethal aerosol doses of BoNT/A and BoNT/B challenge 48 h after mAb administration. Using a novel mAb specific assay based on recombinant mAb specific BoNT domains, we were able to measure the serum concentration of each of the three mAbs comprising NTM-1631 and NTM-1632. Protective levels of NAC were achieved within 6 h post administration of NTM-1631 and NTM-1632 and 100% of animals were completely protected against BoNT challenge. This compares favorably to the approximately 80% of humans that generated protective NAC levels above the lower limit of detection to BoNT/A and BoNT/B after vaccination with a bivalent serotype A and B vaccine based on the H_C_ domain (rBV A/B) [36]. The difference is that active vaccination takes months to achieve protection, thus eliminating the possibility of post-exposure vaccination while NTM-1631 and NTM-1632 could be used post-exposure to provide immediate immunity.

The potential duration of protection in humans against BoNT/A or BoNT/B exposure by NTM-1631 and NTM-1632 can be approximated based on studies on the minimal antibody titer that protects humans against botulism combined with data on the half-lives in humans of mAbs XA-a, XA-b and XA-c from the human Phase 1 trial of NTM-1631 and measurements of the potency of NTM-1631. While the concentration of NAC that protects humans against botulism is unknown, determination of antibody titers likely to be protective in humans have been extrapolated from animal studies. Studies in guinea pigs indicate that serum antitoxin levels of 0.02 U/mL withstood challenge with 200,000 mouse LD_50_s of BoNT and this value was set as the protective level in humans [50]. Separately, Fiock et al. [51] determined protective antibody titers in guinea pigs at 0.02 and 0.005 U/mL for BoNT/A and BoNT/B respectively. As an alternative endpoint for protection in humans, the Centers for Disease Control and Prevention recommended no further vaccine boosts for humans with neutralizing antibody titers greater than 0.25 U/mL [52]. Thus, based on the literature, human protection against botulism would be provided by BoNT antibody titers between 0.02 and 0.25 U/mL for BoNT/A and 0.005 and 0.25 U/mL for BoNT/B. The Phase 1 clinical trial of NTM-1631 showed peak mAb concentrations of 2.35, 2.62 and 2.4 μg/mL for mAbs XA-a, XA-b and XA-c respectively at an injected dose of 0.33 mg/kg [47]. Based on an approximate ED_50_ of 40 U/μg of an equimolar combination of the NTM-1631 mAbs [53], this serum concentration corresponds to a peak serum neutralizing antibody titer of 94 U/mL (2.35 μg/mL × 40 U/μg). From this peak titer, it would take eight half-lives for the NTM-1631 neutralizing antibody titer to reach a serum concentration of 0.367 U/mL (>0.25 U/mL) and twelve half-lives to reach a serum concentration of 0.022 U/mL (>0.02 U/mL). Since the shortest serum half-life of the mAbs comprising NTM-1631, mAb XA-b, was 10.3 days, eight half-lives would correspond to protection for 82 days and twelve half-lives would correspond to protection for 124 days (>4 months). Longer duration of protection could be achieved by using a dose of NTM-1631 or NTM-1632 greater than 0.33 mg/kg. Moreover, duration of protection is likely to be longer than eight to twelve half-lives since guinea pigs with undetectable serum antibody titers (<0.0025 U/mL) have been shown to survive challenge with BoNT and since levels of mAbs XA-a and XA-c would exceed those of mAb XA-b and this mAb pair would provide significant protection [53].

Monoclonal antibodies have become an important class of therapeutics with 87 approved mAbs in clinical use and more than 60 in late stage clinical trials; more than 75% of these mAbs are human or humanized [54]. Besides the mAb combinations for BoNT/A and BoNT/B reported here, we have developed similar combinations for BoNT/C, BoNT/D, BoNT/E [43] and BoNT/F [42]. The BoNT/B, BoNT/C and BoNT/D mAbs have completed Phase 1 clinical testing in humans with no significant adverse events and the BoNT/E mAbs are in Phase 1 testing in humans. The BoNT/F mAbs are in preclinical evaluation. BoNT antitoxins comprised of mAbs may have a number of potential advantages over the existing equine BAT used to treat adult botulism and the human hyperimmune globulin (BIG-IV) used to treat infant botulism. The mAbs comprising NTM-1631 and NTM-1632 and the other mAb combinations are human or humanized and are produced from high expressing CHO-K1 stable cell lines that can be scaled and cGMP manufactured. As such, the recombinant antitoxins are forever renewable from the cell lines, unlike BAT [41] and BIG-IV. The mAbs have the human Fc and thus have a serum half-life in humans of 11–21 days, [47] rather than the 7.5–34 h half-life of BAT. This long half-life precludes the development of relapse of botulism, which has been seen with BAT [55] and as shown here allows for prophylactic administration. While immune responses, including the generation of neutralizing antibodies, can occur with mAbs [56], human and humanized mAbs are thought to be safer than polyclonal serum prepared from immunized animals. While specific experience with NTN-1631 in humans is limited, no significant severe adverse events were seen in the Phase 1 clinical trial of NTM-1631, while both hypersensitivity reactions and serum sickness have been reported for BAT [41,47,57].

For BoNT serotypes A, B, E and F, there exist variants (subtypes) that differ significantly in amino acid sequence and which can result in differences in monoclonal and polyclonal antibody binding and neutralization [42,58]. Selection of mAbs binding conserved epitopes may result in more consistent subtype neutralization that can be achieved using polyclonal serum. For example, a three mAb combination binding all seven BoNT/F subtypes with high affinity provides more than 1000 fold more potent in vivo BoNT/F7 neutralization than BAT [42]. We have not compared the relative potencies of BAT and NTM-1631 and NTM-1632 for BoNT/A or BoNT/B subtypes. However, the three component mAbs of NTM-1631 bind BoNT/A subtypes A1, A2 and A3 with high affinity (K_D_ < 1.0 × 10^−9^ M) and potently neutralize each of these subtypes in mice. Similarly, the component mAbs of NTM-1632 bind BoNT/B subtypes B1, B2, B3 and B4 with high affinity (K_D_ < 1.0 × 10^−9^ M) and potently neutralize each of these subtypes in mice (JDM, unpublished data). Nevertheless, we cannot exclude that there exist BoNT/A or BoNT/B subtypes or engineered BoNTs for which NTM-1631 or NTM-1632 would have reduced potency.

Overall, characteristics in the above two paragraphs support that NTM-1631 and NTM-1632 and the other mAb combinations could be used both for prophylaxis and treatment of both adult and infant botulism, have significant advantages over BAT and BIG-IV and would meet both the Department of Defense’s need for a BoNT/A and BoNT/B vaccine and HHS’ requirement for a multivalent BoNT therapeutic. While BIG-IV is a human polyclonal product with a long serum half-life, the toxoid vaccine used to immunize donors is no longer available and an approved replacement vaccine is not yet available.

## 4. Conclusions

In summary, we have shown how mAb combinations can prevent the development of type A and type B botulism after aerosol exposure in guinea pigs, and postulate that the duration of protection in humans would be long lived based on mAb half-life and the potency of the combinations. Since the mAb combinations can also be used to treat botulism, this holds out the prospect of a single drug for both the treatment and prevention of botulism, uses that cannot be provided by either BIG-IV or BAT.

## 5. Materials and Methods

### 5.1. Monoclonal Antibodies

An oligoclonal mixture of three monoclonal antibodies (mAbs) against BoNT/A, NTM-1631 comprised of an equimolar mixture of mAbs XA-a, XA-b, XA-c, [47] and three mAbs against BoNT/B1, NTM-1632 comprised of an equimolar mixture of mAbs XB-a, XB-b, XB-c [44].

### 5.2. Challenge Material

The BoNT challenge materials were dilutions of the complex form of BoNT/A subtype A1 and BoNT/B subtype B1 purchased from Metabiologics (Madison, WI). The BoNT/A1 was produced from a C. botulinum Hall A strain. The BoNT/B1 was produced from the *C. botulinum* Okra strain. The specific activity of the BoNT/A1 and BoNT/B1 challenge materials were 3.2 × 10^7^ and 7.5 × 10^7^ MIPLD_50_ U/mg protein, respectively [20]. The activity of BoNT for guinea pigs was previously measured at Battelle as one BoNT/A1 GPLD_50_ is equivalent to 158 MIPLD_50_s and one BoNT/B1 GPLD_50_ is equivalent 200 MIPLD_50_s (previously unpublished data). These measured values are in concordance with previously published values of BoNT/A and BoNT/B aerosol LD_50_s in guinea pigs (141 MIPLD_50_s for BoNT/A and 350 MIPLD_50_s for BoNT/B [59]. The research was conducted at a CDC Select Agent Program registered facility and the protocol was reviewed by the local Biosafety Committee.

### 5.3. Control Samples

The BoNT challenge materials and samples for the mouse assays were diluted in 30 mM phosphate buffered saline (PBS, pH 6.2) containing 0.2% (*w*/*v*) gelatin. All aerosol samples were collected in PBS, pH 7.2. The guinea pigs in the animal challenge studies received sterile phosphate buffered saline (PBS), pH 6.5.

### 5.4. Pharmacokinetics

Measurement of antibody circulating concentration post IM or IP dosing was performed prior to the animal challenge studies by using an ECL assay in order to determine the distribution and duration of antibodies in guinea pig circulation and guide dosing for the efficacy studies. Animals were studied in seven cohorts, PBS, NTM-1631 0.06 mg IM, NTM-1631 0.5 mg IM, NTM-1631 1.5 mg IM, NTM-1631 1.5 mg IP, NTM-1632 1.5 mg IM and NTM-1632 1.5 mg IP at ECL assay timepoints of 0, 2, 6, 12, 24, 48, 72 and 120 h and 7, 14, 21, 28, 35, 42 and 70 days using four mice per timepoint. The amount of toxin neutralization afforded by the antibodies (neutralizing antibody concentration, NAC) was assessed separately for each of the seven cohorts using the MNA with BoNT/A1 and BoNT/B1 with concomitant ECL mAb concentration measurements. MNA/ECL assay timepoints were 0, 2, 6, 12, 24, 48 h and 14 day, using 3 mice per timepoint.

The method to measure mAb concentration in guinea pig serum is based on a bridging immunoassay using Meso Scale Discovery (MSD) electrochemiluminescence (ECL) format [47]. The antibody specific domains used are recombinant domains of botulinum toxin A or B [44,45]. Biotinylated and ruthenylated domains were used as the capturing and detecting reagents for the assay, respectively. The assay utilizes the bivalent binding capability of the antibodies to form a bridging complex with biotinylated-domain and ruthenylated-domain to generate ECL signals for the measurement of the target antibody concentration in serum. Six assays were developed using the same format for each antibody assay [47].

Briefly, a solution of reaction mixture containing both biotinylated-domain and ruthenylated-domain plus either calibration standards, assay acceptance controls or study samples was incubated for 1 h at room temperature on an orbital shaker and shielded from light. The calibration standards and assay acceptance controls were prepared using drug products NTM-1631 and NTM-1632 each containing three mAbs XA-a, XA-b and XA-c or XB-a, XB-b and XB-c, respectively [44,45]. The standards were prepared by spiking NTM-1631 or NTM-1632 into neat guinea pig serum to make the highest concentration standard sample at 500 ng/mL (1X assay concentration). The subsequent standard points were prepared by performing 1:2-fold serial dilutions in Sample Dilution Buffer (SDB) until the lowest standard point is made at 0.49 ng/mL, making an 11-point standard calibration curve.

At the end of the initial incubation, the reaction mixtures were transferred to streptavidin plates blocked with Blocking Buffer (BB) and incubated for an additional 1 h at room temperature on the shaker. Antibodies in the standards and study samples formed a bridge between the biotinylated domain and ruthenylated domain and the bridge complex is captured onto the streptavidin coated plates. A chemiluminescence signal was generated when electric current was applied and was detected by the MSD instrument. The resulting signal was measured in ECL units and the concentrations of the antibodies in the assay acceptance controls and study samples were interpolated from the calibration curve.

### 5.5. Animal Challenge Studies

The research was conducted in compliance with the Animal Welfare Act (AWA, 7 U.S.C. §2131, 2002, 2007 and 2008) and other federal statutes and regulations relating to animals and experiments involving animals and adhered to the principles stated in the Guide for the Care and Use of Laboratory Animals (Battelle Biomedical Research Center Protocol Number 3966, approved December 6, 2016). All animal procedures were conducted under protocols approved by the Institutional Animal Care and Use Committees (IACUC) of Battelle Biomedical Research Center, in according with IACUC guidelines, https://www.nal.usda.gov/awic/ institutional-animal-care-and-use-committees. General procedures for animal care and housing were in accordance with the Association for Assessment and Accreditation of Laboratory Animal Care International (AAALAC) recommendations.

For the aerosol challenge studies, a total of 57 male Crl:(HA)Br guinea pigs (*Cavia porcellus*) were used. Animals were randomized into one of four different groups (Table 4).

Two separate experiments were executed with 10 and 100 LD_50_s of BoNT. In each study, each animal received 0.3 mL IM of PBS, NTM-1631 or NTM-1632 per Table 4. The BoNT aerosol challenges were performed approximately 48 h after antibody/PBS administration. In the first aerosol challenge experiment, animals were given a target dose of 10 LD_50_s of either BoNT/A1 or BoNT/B1. The second aerosol challenge experiment involved giving the animals a target dose of 100 LD_50_s of either BoNT/A1 or BoNT/B1. Serum was collected from three animals in each treatment/challenge group on the day of aerosol challenge to determine the NAC at the time of BoNT challenge using the MNA.

### 5.6. Aerosol Exposure System

Guinea pigs were dosed inhalationally, with BoNT using an aerosol delivery system [20,60]. A nose-only aerosol exposure system (CH Technologies, Westwood, NJ, USA) was used to deliver the aerosolized BoNT to each guinea pig. Airflow was regulated using mass flow meters and mass flow controllers to monitor the aerosol flow. Guinea pig respiration rates and minute volumes were calculated by Guyton’s formula [61] using the mean body weight of the animals in each challenge run. The TATV was calculated as the product of the minute volume and the total exposure time.

The amount of BoNT in the aerosols was determined by sampling using a glass impinger (Model 7541, Ace Glass Inc., Vineland, NJ, USA) containing sterile phosphate buffered saline, pH 7.2. The BoNT concentration of the impinger sample was determined using the mouse toxin potency assay described below. The total inhaled dose was then calculated from the product of the aerosol concentration and the TATV. The MIPLD_50_ value for each serotype was then converted to guinea pig LD_50_s using historical values.

(1) $$\mathrm{Inhaled}\;\mathrm{Dose}\;\left({\mathrm{MIPLD}}_{50}\right)=\mathrm{Aerosol}\;\mathrm{Concentration}\;\left(\frac{{\mathrm{MIPLD}}_{50}}{L}\right)\times \mathrm{TATV}\;\left(L\right)$$

Because the size and shape of inhaled aerosols is a critical factor in determining deposition mechanisms and the extent of penetration into the lung and alveolar regions, aerosol particle sizing samples were analyzed using an Aerodynamic Particle Sizer (APS Model 3321, TSI Inc., Shoreview, MN, USA) spectrometer with an aerosol diluter (Model 3302A, TSI Inc.).

### 5.7. Measurement of BoNT Challenge Concentrations

Mouse toxin potency assays were used to determine the BoNT concentration of the BoNT in the impinger and nebulizer samples. Male CD-1 (ICR) mice (*Mus musculus*) purchased from the Charles River Laboratory weighing 18 to 22 g were used for the toxin potency assays. Six concentrations were prepared using a 2-fold dilution factor for each BoNT sample. Five mice per concentration group were injected IP with 0.5 mL of diluted BoNT challenge material. Survival was measured over 96 ± 2 h post-injection. Probit dose-response models were fitted to the dose-lethality data from each challenge dose confirmation using the method of maximum likelihood [62]. Estimated parameters from the probit model were used to estimate LD_50_ dose (MIPLD_50_/mL) for each mouse toxin potency assay. The coefficient of variation (CV) for inter-assay comparisons was 12% for BoNT/A and 7% for BoNT/B (internal data).

### 5.8. Measurement of Neutralizing Antibody Concentration

NACs were determined using a standardized and validated mouse neutralization assay (MNA) based on methods developed by Cardella and Hatheway and Dang [59,63]. Battelle Biomedical Research Center (Columbus, OH, USA) provides the only FDA approved, statistically-validated MNA for determining anti-BoNT antibody concentrations (NACs) in the United States [64]. The NACs in this study were determined by Battelle using identical procedures and reference standards as those used for NAC determinations in the development of BIG-IV and its eventual licensure in 2003 [37,65] and as reported in other publications [64,66]. Male CD-1 (ICR) mice (*Mus musculus*) purchased from the Charles River Laboratory and weighing 17 to 23 g were used for the MNA. A standard curve assay containing nine square-root of two dilutions of antitoxin reference standards was prepared for each set of guinea pig serum samples tested. The reference standards were PI-A, Botulinum Immune Globulin, F(ab’)_2_, Heptavalent, Equine, Lot AAT manufactured by PerImmune, Inc. (Rockville, MD, USA) and calibrated in Units (1 U = 10,000 mouse IP LD_50_s/mL for BoNT/A and WHO-B, Lot 13209 3000 prepared and characterized by the Statens Serum Institut (Copenhagen, Denmark) and expressed in IU/mL (where IU = 10,000 mouse IP LD_50_s/mL) for BoNT/B. Seven four-fold dilutions were prepared for each serum sample. The antitoxin reference standards and guinea pig serum samples were titrated against a fixed amount of BoNT [44 mouse LD_50_/mL for BoNT/A1 and 25 mouse LD_50_/mL for BoNT/B1), incubated for 60 to 120 min at room temperature and 0.2 mL was injected IP into mice. The BoNT/A1 and BoNT/B1 challenge concentrations were based on calibration experiments performed to determine the concentration that was 50% neutralized by 0.02 U/mL of the PerImmune-A and 0.005 U/mL WHO-B antitoxin standards, respectively. Survival was measured over 96 ± 2 h post-injection. To determine the ED_50_ for the antitoxin standards and serum samples, a probit dose response curve was fitted to the lethality results as a function of the base 10 logarithm of antibody concentration or serum sample dilution. The NAC was calculated as the ratio of the ED_50_ of the standard curve, over the ED_50_ of the test curve.

### 5.9. Statistical Methods

Probit dose-response models were fitted to the dose-lethality data from each challenge dose confirmation in mice using the method of maximum likelihood [62]. Estimated parameters from the probit model were used to estimate LD_50_ dose (MIPLD_50_/mL) for each mouse toxin potency assay. For each MNA, probit analysis was used to fit a dose-response curve to the proportion of mice dead as a function of the base 10 logarithm of the antibody concentration. The NAC for each sample was estimated from the probit curves for the samples and associated reference standard. The ED_50_ of the associated standard was divided by the ED_50_ for the assay corresponding to the test sample. For the animal challenge studies, survival rates in treated and control groups were compared using the two-sided 0.05-level Boschloo tests.

To test the hypothesis that individual antibodies within NTM-1631 or NTM-01632 had the same or different concentrations, we performed repeated-measures two-way analysis of variance (ANOVA) with time as a within-subject factor and antibody as a between-subject factor. Variance was assumed to be equal for all antibodies. When ANOVA identified there was a difference, we used a *t*-test with the Holm-Šídák correction for multiple comparisons to identify which antibodies and time points had different concentrations. Antibody concentrations were assumed to have the same standard deviation. Calculations were performed using Prism v 6.0, (GraphPad Software Inc., La Jolla, CA, USA, 2014) 

## Figures and Tables

**Table 1:** Summary of botulinum neurotoxin (BoNT) Aerosol Exposure Parameters.

Parameters		Study 1		Study 2	
		BoNT/A1	BoNT/B1	BoNT/A1	BoNT/B1
Guinea Pig LD_50_ delivered	Target	10	10	100	100
	Actual	55	8	93	116
Inhaled Dose (MIPLD_50_/animal)	Actual	8.73 × 10^3^	1.52 × 10^3^	1.47 × 10^4^	2.33 × 10^4^
Total accumulated tidal volume (TATV) (L)	Actual	2.32	2.38	2.43	2.40
Impinger Concentration (MIPLD_50_/mL) *	Actual	1.39 × 10^4^	2.34 × 10^3^	2.26 × 10^4^	3.54 × 10^4^
Nebulizer Suspension Concentration (MIPLD_50_/mL) *	Actual	1.04 × 10^6^	4.67 × 10^5^	2.00 × 10^6^	4.77 × 10^6^
Aerosol Conc. (MIPLD_50_/L) *	Actual	3.76 × 10^3^	6.39 × 10^2^	6.07 × 10^3^	9.69 × 10^3^
Mass median aerodynamic diameter (µm)	Actual	1.16	1.05	1.15	1.12
Exposure Time (min)	Actual	12.00	12.00	12.00	12.00
# Animals/Exposure	Actual	20	20	20	20
Mean Animal Weight (g)	Actual	417	431	441	435

* MIPLD_50_ = mouse intraperitoneal LD_50_s.

**Table 2:** BoNT/A and BoNT/B Neutralizing Antibody Concentrations (NAC) administered IM.

Treatment Group	Study	Geometric Mean NAC (U/mL)	95% Confidence Bounds
1-PBS	1	NM	NA
	2	NM	NA
2-BoNT/A1	1	7.7	(4.5, 13.1)
	2	9.7	(1.1, 83)
1-PBS	1	NM	NA
	2	NM	NA
2-BoNT/B1	1	21.6	(9.9, 47.3)
	2	19.5	(5.7, 65.6)

NM, no measurable NAC levels; NA, not applicable.

**Table 3:** Guinea Pig Challenge Time to Death and Mortality per Group.

Treatment	mAb Dose (mg)	BoNT Challenge Serotype	Average Time to Death (h)	Average Weight Gain after 14 Days (g)	Mortality (Number Dead/Total Number Animals)
	First Challenge Experiment				
PBS	0	BoNT/A1 (55 LD_50_)	15	N/A	10/10
NTM-1631	1.5		N/A	164.67	0/10
PBS	0	BoNT/B1 (8 LD_50_)	24	N/A	10/10
NTM-1632	1.5		N/A	178.88	0/10
	Second Challenge Experiment				
PBS	0	BoNT/A1 (93 LD_50_)	13	N/A	10/10
NTM-1631	1.5		N/A	100.74	0/10
PBS	0	BoNT/B1 (116 LD_50_)	14	N/A	10/10
NTM-1632	1.5		N/A	97.17	0/10

**Table 4:** Aerosol challenge in Guinea Pig experimental design.

Treatment Group	mAb Concentration (mg)	Number of Animals Per Group *	Challenge Material
PBS	0	13	BoNT/A1
NTM-1631	1.5	13	BoNT/A1
PBS	0	13	BoNT/B1
NTM-1632	1.5	13	BoNT/B1

* Serum was collected from three animals 48 h post-dose of mAbs. The remaining 10 animals went on the challenge timeline.

## Supplementary Materials

The following are available online at https://www.mdpi.com/2072-6651/11/4/208/s1, Table S1: Neutralizing Antibody Concentrations (NAC) of NTM-1631 in individual guinea pigs. Table S2: Neutralizing Antibody Concentrations (NAC) of NTM-1632 in individual guinea pigs. Table S3: Raw data of mouse neutralization assay used to determine individual guinea pig NAC reported in Table S1. Table S4: Raw data of mouse neutralization assay used to determine individual guinea pig NAC reported in Table S2.
