# Supplementary material for: Monoclonal Antibody Combinations Prevent Serotype A and Serotype B Inhalational Botulism in a Guinea Pig Model
Source: Toxins (Basel). 2019 Apr 6;11(4):208. doi: 10.3390/toxins11040208 (PMC6520708; doi:10.3390/toxins11040208)
Supplement: Supplementary file 1 [file toxins-11-00208-s001.pdf]

Supplementary Materials: Monoclonal Antibody Combinations Prevent Serotype A and Serotype B Inhalational Botulism in a Guinea Pig Model

Milan T. Tomic *, Yero Espinoza, Zachary Martinez, Khanh Pham, Ronald R. Cobb, Doris M. Snow, Christopher G. Earnhart, Traci Pals, Emily S. Syar, Nancy Niemuth, Dean J. Kobs, Shauna Farr-Jones and James D. Marks

Table 1. Neutralizing Antibody Concentrations (NAC) of NTM-1631 in individual guinea pigs.

Group	Study Iteration	Guinea Pig ID Number	NAC (Units/mL)
PBS	1	223	NM
		255	NM
		229	NM
NTM-1631	1	235	8.7
		217	8.7
		208	6.0
PBS	2	294	NM
		290	NM
		286	NM
NTM-1631	2	272	25
		259	7.9
		287	4.6

NTM-1631 delivered intramuscularly, 1.5 mg dose. Two study iterations were performed. N.M. = No measurable levels.

Table S2. Neutralizing Antibody Concentrations (NAC) of NTM-1632 in individual guinea pigs.

Treatment Group	Study Iteration	Guinea Pig ID Number	NAC (Units/mL)
PBS	1	218	NM
		219	NM
		220	NM
NTM-1632	1	231	16
		249	21
		224	30
PBS	2	279	NM
		264	NM
		296	NM
NTM-1632	2	310	12
		266	32
		281	19

NTM-1632 delivered intramuscularly, 1.5mg dose. Two study iterations were performed. NM = Not measurable.

Table S3. Raw data of mouse neutralization assay used to determine individual guinea pig NAC reported in Table S1. Testing was completed in two iterations using PerImmune BoNT/A/09/97 reference antitoxin (PI-A).

Treatment	Guinea Pig ID		Antibody Concentration (Serial Dilution)	No. Mice Dead/ Number Tested	
	Study 1	Study 2		Study 1	Study 2
PBS				0/7	0/7
PI-A (no toxin)				0/7	0/7
BoNT/A1 (no antibody)				6/7	6/7
PI-A	(No Guinea Pig)	(No Guinea Pig)	0.005	6/7	6/7
			0.0071	7/7	7/7
			0.01	7/7	6/7
			0.014	6/7	6/7
			0.02	3/7	2/7
			0.028	0/7	0/7
			0.04	0/7	0/7
			0.057	0/7	0/7
			0.08	0/7	0/7
PBS	223	294	1	7/7	7/7
			0.25	7/7	7/7
			0.063	7/7	7/7
			0.016	6/7	7/7
			0.0039	7/7	5/7
			0.00098	6/7	5/7
			0.00024	7/7	6/7
PBS	255	290	1	6/7	7/7
			0.25	7/7	6/7
			0.063	6/7	7/7
			0.016	6/7	6/7
			0.0039	7/7	7/7
			0.00098	7/7	7/7
			0.00024	6/7	7/7
PBS	229	286	1	7/7	7/7
			0.25	7/7	6/7
			0.063	6/7	6/7
			0.016	6/7	7/7
			0.0039	7/7	7/7
			0.00098	7/7	7/7
			0.00024	7/7	6/7
NTM-1631	235	272	0.25	0/7	0/7
			0.063	0/7	0/7
			0.016	0/7	0/7
			0.0039	1/7	0/7
			0.00098	6/7	5/7
			0.00024	7/7	7/7
			0.000061	7/7	4/7
NTM-1631	217	259	0.25	0/7	0/7
			0.063	0/7	0/7
			0.016	0/7	0/7
			0.0039	0/7	0/7
			0.00098	7/7	7/7
			0.00024	7/7	7/7
			0.000061	7/7	7/7

			0.25	0/7	0/7
			0.063	0/7	0/7
			0.016	0/7	0/7
NTM-1631	208	287	0.0039	3/7	1/7
			0.00098	6/7	7/7
			0.00024	7/7	7/7
			0.000061	7/7	7/7

Table S4. Raw data of mouse neutralization assay used to determine individual guinea pig NAC reported in Table S2. Testing was completed in two iterations using WHO BoNT/B 13209 3000.1185 reference antitoxin (WHO-B).

	**Guinea Pig ID**			**No. Mice Dead / Number Tested**	
**Treatment**	**Study 1**	**Study 2**	**Antibody Concentration**	**Study 1**	**Study 2**
PBS	(No guinea pig)	(No guinea pig)	N.A.	0/7	0/7
WHO-B (Control)			N.A.	0/7	0/7
BoNT/B1 (Control)			N.A.	5/7	7/7
WHO-B			0.0013	7/7	7/7
			0.0018	5/7	6/7
			0.0025	6/7	6/7
			0.0035	7/7	3/7
			0.005	2/7	3/7
			0.0071	0/7	0/7
			0.01	0/7	0/7
			0.014	0/7	0/7
			0.02	0/7	0/7
PBS	279	218	1	7/7	7/7
			0.25	6/7	7/7
			0.063	7/7	7/7
			0.016	6/7	7/7
			0.0039	7/7	6/7
			0.00098	6/7	5/7
			0.00024	6/7	7/7
PBS	264	219	1	7/7	7/7
			0.25	7/7	7/7
			0.063	7/7	7/7
			0.016	6/7	7/7
			0.0039	6/7	7/7
			0.00098	7/7	7/7
			0.00024	7/7	5/6
PBS	296	220	1	7/7	7/7
			0.25	6/7	7/7
			0.063	7/7	6/7
			0.016	6/7	7/7
			0.0039	6/7	7/7
			0.00098	6/7	6/7
			0.00024	6/7	7/7

Table S4. *Cont.*

			0.015	0/7	0/7
			0.0038	0/7	0/7
			0.00096	0/7	0/7
NTM-1632	310	231	0.00024	7/7	6/7
			0.00006	5/7	6/7
			0.000015	6/7	7/7
			0.0000038	7/7	7/7
			0.015	0/7	0/7
			0.0038	0/7	0/7
			0.00096	0/7	0/7
NTM-1632	266	249	0.00024	5/7	1/7
			0.00006	7/7	7/7
			0.000015	7/7	6/7
			0.0000038	5/7	7/7
			0.015	0/7	0/7
			0.0038	0/7	0/7
			0.00096	0/7	0/7
NTM-1632	281	224	0.00024	3/7	5/7
			0.00006	7/7	7/7
			0.000015	6/7	5/7
			0.0000038	6/7	7/7
